# James Williams Arrowsmith

**Published:** 1913-03

**Authors:** 


### JAMES WILLIAMS ARROWSMITH.

When thirty years ago Mr. Greig Smith and Mr. L. M. Griffiths were arranging for the establishment of a Journal of the Medical Sciences for the West of England and South Wales, they naturally appealed to Mr. Arrowsmith, the publisher of Mr. Greig Smith's pioneer work on *Abdominal Surgery*. He at once saw the wisdom of their proposal, and gave every possible assistance and advice as to the details of the business part of the undertaking.

The Medico-Chirurgical Society undertook the financial responsibility, and the *Journal* then established has continued to run a prosperous course, bringing during its early years a fairly large profit, which was reserved, and afterwards devoted to the formation of the Medical Library.

It is due to our late publisher, who died on January 19th, in his seventy-fourth year, to state that he was always greatly interested in the welfare of the *Journal*, and anxious for its success. We were fortunate in obtaining the assistance of one whose whole heart was in the work.

The address given by the Rev. Dr. Glover at the funeral on January 23rd brought out the many-sided character of Mr. Arrowsmith's life, which appeared to include in one personality the lives of three or four different men. The many sides have been commented on by the usual Press notices, but there is one which has been left us to indicate, and we make no apology for a short eulogy of one who did so much for the medical profession, and was so largely concerned in the establishment of our *Journal*. For many years he was an active supporter of the University College, with which was affiliated and afterwards incorporated the old Bristol Medical School. He shared with the medical staff in the feeling that the Bristol student was not quite fairly treated, and could not be until a Bristol University became something more than an aspiration or a dream of the future. How he worked towards the attainment of this end is well known to all men, and his perseverance was at last rewarded by the establishment of what had been for many years the highest aim of his ambition. The medical profession is greatly indebted to him for the fact that the Bristol student is now no longer at any disadvantage in obtaining a University degree on similar terms to those which exist elsewhere.

